# Highly Pathogenic Porcine Reproductive and Respiratory Syndrome Virus, Asia

**DOI:** 10.3201/eid1709.110411

**Published:** 2011-09

**Authors:** Tong-Qing An, Zhi-Jun Tian, Chao-Liang Leng, Jin-Mei Peng, Guang-Zhi Tong

**Affiliations:** Author affiliations: State Key Laboratory of Veterinary Biotechnology, Harbin Veterinary Research Institute, Harbin City, People’s Republic of China (T.-Q. An, Z.-J. Tian, C.-L. Leng, J.-M. Peng, G.-Z. Tong);; Shanghai Veterinary Research Institute, Shanghai, People’s Republic of China (G.-Z. Tong)

**Keywords:** highly pathogenic porcine reproductive and respiratory syndrome virus, PRRSV, porcine reproductive and respiratory syndrome, Asia, risk, viruses, letter

**To the Editor:** Recently, the novel and highly virulent variant of porcine reproductive and respiratory syndrome virus (PRRSV), which first emerged in the People’s Republic of China and Vietnam in 2006 (*1*), has rapidly spread in pigs in Southeast Asia. The affected countries include Bhutan, Cambodia, Laos, Malaysia, Myanmar, the Philippines, Thailand, and Singapore. In eastern and northern Asia, South Korea and Russia were also reported to be affected (*2*) (Figure). The epidemic affected not only large commercial farms but also the backyard industry, which created a serious problem for the global swine industry and for food safety. In February 2011, the Veterinary and Animal Breeding Agency in Ulaanbaatar, Mongolia, confirmed an outbreak of porcine reproductive and respiratory syndrome (PRRS) (*3*). Nearby neighbors, such as Japan, North Korea, Indonesia, and other Asia-Pacific countries, are also at risk.

**Figure:**
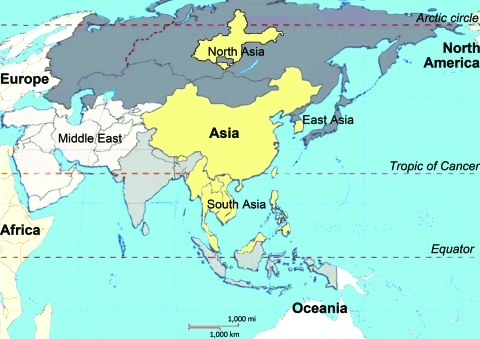
Areas in Asia where outbreaks of highly pathogenic porcine reproductive and respiratory virus syndrome occurred. The countries or regions affected (North Asia, East Asia, Asia, and South Asia) are indicated.

PRRS was first reported in the United States in 1987. The disease causes reproductive failure during late-term gestation in sows and respiratory disease in pigs of all ages. In 2006, a new, highly pathogenic PRRS emerged, characterized by high fever (41°C–42°C), skin discoloration/reddening, high incidence of illness (50%–100%), and high proportion of deaths (20%–100%) in pigs of all ages. This new PRRS has spread throughout the swine industry in China, resulting in the culling of an estimated 20 million pigs annually in 2006–2007 in China (*4*). PRRSV is a member of the family *Arteriviridae* in the order Nidovirales, which also includes severe acute respiratory syndrome coronavirus.

PRRSV is a single-stranded positive sense RNA virus that shows high rates of genetic diversity. In the genome of the novel highly pathogenic PRRSV mutant, 4 deletions (2 deletions in nonstructural protein 2, one deletion in the 5′ untranslated region, and one deletion in the 3′ untranslated region), and some other point mutations, have occurred, which were markedly different from those found in any other previous virus isolate.

After a surveillance study of the epidemic and an analysis of >300 novel highly pathogenic PRRSVs were conducted, the highly pathogenic PRRSV from China was considered to have gradually evolved from CH-1a, a local PRRSV isolate. The evolutionary path could be traced through intermediate PRRSV strains (*5*). Moreover, we found that highly pathogenic PRRSV has a further enlarged deletion in nonstructural protein 2.

Highly pathogenic PRRSV first emerged in China and Vietnam almost simultaneously in 2006, and the epidemic focus was in the area between southern China and northern Vietnam (*6**,**7*). Although no evidence has shown that the highly pathogenic PRRSV isolate from China or Vietnam has spread in other areas, highly pathogenic PRRS has spread throughout the Malaysian Peninsula to southern Russia.

In addition, all highly pathogenic PRRSV isolates share high sequence identity and have the same deletions as the highly pathogenic PRRSV isolated from China or Vietnam. PRRSV can spread through a variety of routes, including direct contact between pigs, droplet contact through nasal secretions, direct contact with saliva and feces, and indirect contact.

PRRS has spread rapidly around the world through pig sales, semen, and airborne transmission, including from airline passengers who carry the virus on their clothing, shoes, or equipment while traveling (*8*). In the global market, any virus emerging in the highly pathogenic form is a threat. The risk of highly pathogenic PRRS spreading to other countries is increasing.
